# Maternal Quality of Life Following a Periviable Delivery

**DOI:** 10.31486/toj.25.0008

**Published:** 2025

**Authors:** Juliann Wang, Christina Blanchard, Angela Seasely, Abigail Cooley, Danyon Beitel, Colm Travers, Brian Sims, Alan Tita, Brian Casey, Rachel Sinkey

**Affiliations:** ^1^ University of Alabama at Birmingham Heersink School of Medicine, Birmingham, AL; ^2^ Department of Obstetrics and Gynecology, University of Alabama at Birmingham, Birmingham, AL; ^3^ Center for Research in Women's Health, University of Alabama at Birmingham, Birmingham, AL; ^4^ Department of Pediatrics, Division of Neonatology, University of Alabama at Birmingham, Birmingham, AL; ^5^ University of Alabama at Birmingham School of Public Health, Birmingham, AL

**Keywords:** *Maternal health*, *premature birth*, *quality of life*

## Abstract

**Background:**

Data on long-term maternal quality of life following a periviable delivery are lacking. We investigated quality of life measures among patients who delivered a periviable infant.

**Methods:**

We conducted a retrospective cohort study of patients who delivered between gestational ages 22^0/7^ to 25^6/7^ weeks at a single institution from 2013 to 2019 and who completed the validated World Health Organization Quality of Life–Brief Version (WHOQOL–BREF) questionnaire between 2021 and 2022. Mothers who had a surviving infant discharged from the hospital were compared with mothers who had an intrauterine or neonatal demise. Baseline characteristics were summarized, and scores were evaluated based on the 4 prespecified WHOQOL–BREF domains: physical health, psychological health, social relationships, and environment.

**Results:**

Overall, 58 patients participated: 32 mothers with a surviving infant and 26 mothers with a demise. Maternal age was lower in the mothers with a surviving infant (25.2 ± 3.9 years vs 29.0 ± 6.0 years; *P*=0.01). Mean duration from delivery to study start date was not different between the groups (5.1 ± 2.0 years vs 4.5 ± 2.1 years; *P*=0.31). Mothers with surviving infants scored higher (indicating higher quality of life) in the WHOQOL–BREF psychological health and social relationships domains compared to mothers with a demise. Mothers with a surviving infant were more likely to be satisfied with their own health than mothers with a demise, but overall quality of life was not different between the 2 groups.

**Conclusion:**

In this small cohort, mothers with an infant who survived a periviable delivery, despite possible long-term health complications for the infant, reported improved quality of life in some domains compared to mothers whose infants did not survive.

## INTRODUCTION

The American College of Obstetricians and Gynecologists defines periviable birth as a delivery that occurs between 20^0/7^ and 25^6/7^ weeks’ gestation.^1^ The parents’ decision of whether or not to resuscitate and provide ongoing care to a periviable neonate is multifactorial and challenging. Many infants delivered between 22 and 24 weeks’ gestation will die during the neonatal period or experience long-term morbidity.^2,3^

Periviable pregnancy management includes assessment of maternal and fetal status, as well as the risk of mortality and morbidity within the context of the parents’ goals of care, in an effort to optimize shared decision-making.^4^ Maternal counseling at the threshold of fetal viability often must carefully balance the likelihood of neonatal survival with maternal risk and future pregnancy implications. For example, the decision of whether to elect a classic cesarean section during the periviable period—which is associated with maternal blood loss and infection and requires future early-term cesareans—is an extremely personal choice with both short- and long-term consequences.

Few studies have investigated the long-term effects that infant outcomes following periviable delivery have on the mother's quality of life. A prior study examined the impact of very low birth weight infants on maternal quality of life through infancy and into early childhood,^5^ but literature on this topic is lacking. To investigate the quality of life among patients who delivered a periviable infant at our institution, we used the validated World Health Organization Quality of Life–Brief Version (WHOQOL–BREF)^6^ questionnaire (Appendix).

## METHODS

We conducted a retrospective cohort study of mothers who delivered a periviable infant at the University of Alabama at Birmingham (UAB) Hospital, a tertiary hospital in Birmingham, Alabama, that serves as a statewide referral center with a Level IV neonatal intensive care unit able to offer advanced resuscitation including extracorporeal membrane oxygenation. The UAB Institutional Review Board approved the research (IRB-300002628).

Patients were identified by a query of the Obstetric Automated Records database and were included if the individual delivered between 22^0/7^ and 25^6/7^ weeks’ gestation at UAB Hospital from 2013 to 2019. Mothers who died, mothers with infants diagnosed with major congenital anomalies, and non-English-speaking mothers were excluded from the study.

Mothers of infants were categorized into 2 groups: those who had a surviving infant discharged from the hospital and those who had an intrauterine or neonatal demise. From September 2021 to May 2022, trained team members (JW, AC, DB, and AS) called mothers up to 3 times to invite them to participate in the study. Baseline maternal and neonatal characteristics were identified by medical records review, and duration of time from delivery to study start date was calculated. Maternal baseline characteristics were maternal age at delivery, self-reported race, insurance status, marital status, prenatal care utilization, parity, body mass index at delivery, chronic hypertension, and preeclampsia. Neonatal outcomes were gestational age at delivery, bronchopulmonary dysplasia, pulmonary hypertension, hypertonicity concerning for cerebral palsy/developmental delay, sepsis, intraventricular hemorrhage, necrotizing enterocolitis, retinopathy of prematurity, respiratory support at discharge, and survival to 1 year of life.

The primary outcome was quality of life scores, assessed via the validated WHOQOL–BREF questionnaire,^6^ between mothers who had a surviving infant discharged from the hospital and mothers with intrauterine or neonatal demise. Scores for the primary outcome were evaluated based on the 4 prespecified WHOQOL–BREF domains of physical health, psychological health, social relationships, and environment. We conducted a secondary analysis to assess differences in responses to individual questions. Domain and individual question scores are scaled in a positive direction; higher scores indicate higher quality of life. A scoring guide with a description of which questions contribute to each domain is available at the WHOQOL–BREF website.^6^

Using SAS version 9.4 (SAS Institute Inc), we compared baseline characteristics and outcomes between groups using *t* tests and Wilcoxon rank sum tests for continuous variables and chi-square tests and Fisher exact tests for categorical variables. The level of significance for all analyses was set at *P*<0.05.

## RESULTS

A total of 606 eligible patients were identified, but 548 patients could not be reached or declined to participate. A total of 58 patients volunteered to participate in the study: 32 mothers with a surviving infant at hospital discharge and 26 mothers with an intrauterine or neonatal demise. Maternal characteristics including race, insurance status, marital status, and mode of delivery were not different between groups. However, mothers with a surviving infant were younger (25.2 ± 3.9 years vs 29.0 ± 6.0 years; *P*<0.01) and less likely to have chronic hypertension (3.1% vs 23.1%; *P*=0.04) or preeclampsia (6.3% vs 34.6%; *P*=0.01) than mothers with a demise (Table 1). The mean duration from delivery to study start date was similar between cohorts (5.1 ± 2.0 years vs 4.5 ± 2.1 years; *P*=0.31); gestational age at delivery was also similar between groups (24.1 ± 0.9 vs 23.8 ± 1.3 weeks; *P*=0.40).

**Table 1.** Baseline Characteristics for Mothers With a Periviable Delivery

	Periviable Delivery		
Variable	Surviving Infant, n=32	Infant Demise, n=26	*P* Value
Maternal age at delivery, years, mean ± SD	25.2 ± 3.9	29.0 ± 6.0	0.01
Race			0.56
African American	19 (59.4)	18 (69.2)	
Caucasian	11 (34.4)	8 (30.8)	
Asian	2 (6.3)	0 (0.0)	
Insurance status			0.52
Medicaid/self-pay	15 (46.9)	10 (38.5)	
Private	17 (53.1)	16 (61.5)	
Marital status			0.51
Married	20 (62.5)	14 (53.8)	
Single	12 (37.5)	12 (46.2)	
No prenatal care	16 (50.0)	11 (42.3)	0.56
Multiparous	13 (40.6)	14 (53.8)	0.32
Body mass index at delivery, kg/m^2^, mean ± SD	35.9 ± 11.5	35.6 ± 11.3	0.92
Chronic hypertension	1 (3.1)	6 (23.1)	0.04
Preeclampsia	2 (6.3)	9 (34.6)	<0.01
Gestational age at delivery, weeks, mean ± SD	24.1 ± 0.9	23.8 ± 1.3	0.40
Mode of delivery			0.27
Vaginal	15 (46.9)	16 (61.5)	
Cesarean	17 (53.1)	10 (38.5)	
Duration from delivery to study start date, years, mean ± SD	5.1 ± 2.0	4.5 ± 2.1	0.31

Note: Data are presented as n (%) unless otherwise indicated.

Complications in surviving infants included bronchopulmonary dysplasia (96.9%); need for respiratory support at discharge (56.3%); pulmonary hypertension (28.1%); culture-positive sepsis (37.5%); intraventricular hemorrhage (43.8%), with 29% classified as grade 3 or 4; necrotizing enterocolitis (15.6%); retinopathy of prematurity (84.4%); retinopathy of prematurity requiring treatment (31.3%); and hypertonicity concerning for cerebral palsy/developmental delay (31.3%). Ninety-one percent of survivors remained alive at 1 year of life.

**Figure 1.**
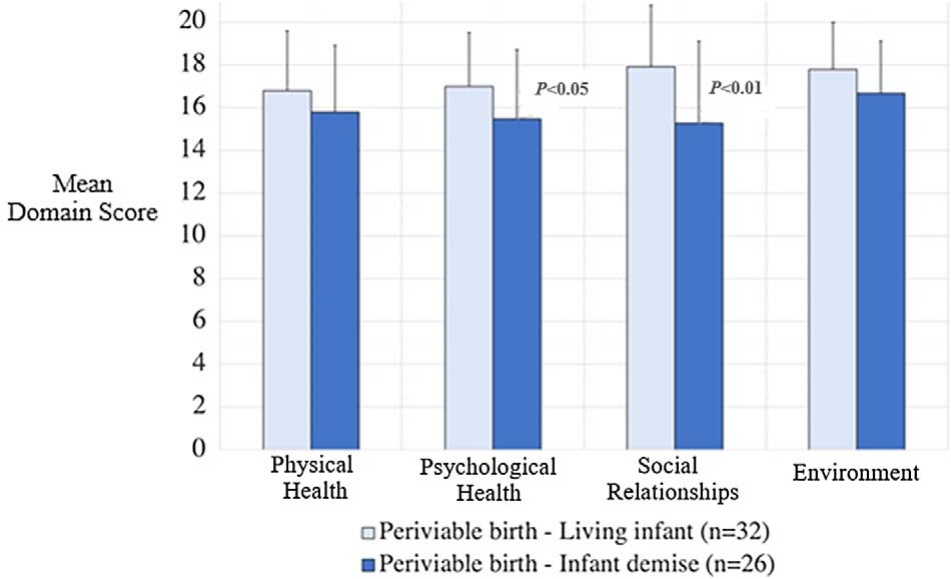
World Health Organization Quality of Life–Brief Version (WHOQOL–BREF) questionnaire domain scores. Scores are scaled in a positive direction; higher scores indicate higher quality of life.

In the primary outcome assessment of the 4 prespecified WHOQOL–BREF domains, mothers with surviving infants scored higher (indicating a higher quality of life) in the psychological health (17.0 ± 2.5 vs 15.5 ± 3.2; *P*<0.05) and social relationships (17.9 ± 2.9 vs 15.3 ± 3.8; *P*<0.01) domains compared to mothers who had a demise (Table 2 and Figure 1). No differences between groups were identified in the physical health (*P*=0.18) or environment (*P*=0.08) domains.

**Table 2.** World Health Organization Quality of Life–Brief Version (WHOQOL–BREF) Questionnaire Scores for Mothers With a Periviable Delivery

	Periviable Delivery		
Variable	Surviving Infant, n=32	Infant Demise, n=26	*P* Value
**Domains**			
Physical health	16.8 ± 2.8	15.8 ± 3.1	0.18
Psychological health	17.0 ± 2.5	15.5 ± 3.2	<0.05
Social relationships	17.9 ± 2.9	15.3 ± 3.8	<0.01
Environment	17.8 ± 2.2	16.7 ± 2.4	0.08
**Select Individual Questions**			
How satisfied are you with your health?	4.3 ± 0.7	3.8 ± 0.8	0.02
How would you rate your quality of life?	4.6 ± 0.5	4.2 ± 0.9	0.08
How much do you enjoy life?	4.6 ± 0.8	4.2 ± 0.8	0.06
To what extent do you feel your life to be meaningful?	4.7 ± 0.6	4.5 ± 0.9	0.42
Have you enough money to meet your needs?	4.3 ± 1.1	3.6 ± 1.4	0.02
How satisfied are you with your sleep?	3.9 ± 1.2	3.1 ± 1.3	0.02
How satisfied are you with your personal relationships?	4.5 ± 0.8	4.0 ± 1.0	0.05
How satisfied are you with your sex life?	4.5 ± 0.8	3.7 ± 1.4	0.03
How satisfied are you with the support you get from your friends?	4.5 ± 0.9	3.8 ± 1.3	0.03
How often do you have negative feelings such as blue mood, despair, anxiety, depression?	2.5 ± 1.2	3.0 ± 1.2	0.12

Notes: Data are presented as mean ± SD. Domain and individual question scores are scaled in a positive direction; higher scores indicate higher quality of life. The maximum score possible for each individual question was 5; the maximum score possible for each domain was 20.

**Figure 2.**
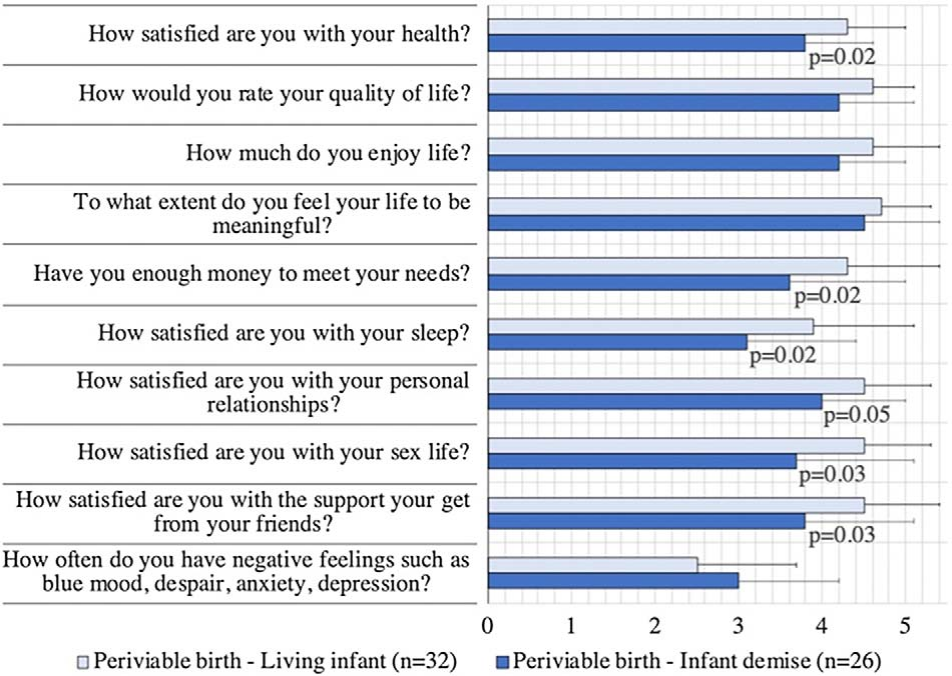
World Health Organization Quality of Life–Brief Version (WHOQOL–BREF) questionnaire select individual question scores. Scores are scaled in a positive direction; higher scores indicate higher quality of life.

In the assessment of individual questions, mothers with a surviving infant were more likely to express higher satisfaction with their own health (4.3 ± 0.7 vs 3.8 ± 0.8; *P*=0.02) than mothers with a demise (Table 2 and Figure 2). Mothers with a surviving infant were also more likely to report having enough money to meet their needs (4.3 ± 1.1 vs 3.6 ± 1.4; *P*=0.02) and to be satisfied with their sleep (3.9 ± 1.2 vs 3.1 ± 1.3; *P*=0.02), sex life (4.5 ± 0.8 vs 3.7 ± 1.4; *P*=0.03), and support from friends (4.5 ± 0.9 vs 3.8 ± 1.3; *P*=0.03) compared to mothers with a demise. However, we found no difference in responses to questions asking participants to rate their quality of life (*P*=0.08), their enjoyment of life (*P*=0.06), the extent they felt their life to be meaningful (*P*=0.42), their satisfaction with personal relationships (*P*=0.05), and their experiences with negative feelings (*P*=0.12).

## DISCUSSION

Periviable infants who survive delivery have an increased risk of morbidity, health care utilization, and death.^1,2^ However, data from our small cohort show that despite the possible long-term health complications, mothers with an infant who survived a periviable delivery reported higher quality of life scores in some domains. This study demonstrates the importance of evaluating measures other than morbidity and mortality when examining the effect periviable deliveries have on mothers. In doing so, providers may be able to offer a more holistic approach to maternal care.

Despite the growing body of research examining the effects of preterm birth on maternal mental health,^7^ investigation into maternal quality of life following periviable delivery is limited. Prior studies have compared psychological distress and mental health problems between parents of children born very preterm and parents of children born at full term,^8^ with little attention to the challenges and experiences of mothers who delivered infants at the threshold of viability. More comprehensive understanding of the experiences and factors that impact maternal quality of life can improve support and care for these mothers and their families.

This study has several limitations. First, the sample size (n=58) is small. Consequently, this cohort may be skewed, may not be representative of the broader population, and thus may limit generalizability. Additionally, the responses of individuals who declined participation or whom we could not contact could potentially have been very different than the responses of those included in the study. A study by Tucker Edmonds et al showed that decisions about perinatal care impacted mental health outcomes for families,^9^ but we did not assess whether the mothers of infants who died made the decision to provide or withdraw perinatal care prior to demise. Moreover, we did not investigate connections between specific neonatal outcomes and maternal quality of life. Finally, participants’ lives may include additional confounding factors that could impact scores but that are not captured by the validated questionnaire, such as the use of counseling services or medications aimed at supporting quality of life.

An important limitation of this study that should be addressed in future studies is to standardize the time from delivery to survey completion. In our cohort, patients delivered between 2013 and 2019 and were contacted between 2021 and 2022. In future studies, patients should be compared within a consistent time range following delivery to reduce potential confounding.

All of these factors beg the question: what difference in a quality of life score is meaningful? While the WHOQOL–BREF is a validated questionnaire, an individual's quality of life is extremely personal and based on individual preferences, values, culture, and priorities. Future prospective studies should explore these individual factors and whether pregnancy outcomes influence overall quality of life.

## CONCLUSION

In terms of patient-reported outcomes, these data demonstrate an important first step in providing insight into the mother's perspective after a periviable delivery. Further prospective investigation is essential to understand specific factors that impact maternal well-being in these sensitive situations.

## Notes

*This article meets the Accreditation Council for Graduate Medical Education and the American Board of Medical Specialties Maintenance of Certification competencies for Patient Care, Medical Knowledge, and Practice-Based Learning and Improvement.*

## Appendix. World Health Organization Quality of Life–Brief Version (WHOQOL–BREF) Questionnaire

*Reproduced with permission from WHOQOL–BREF: introduction, administration, scoring and generic version of the assessment: field trial version, December 1996, Geneva, World Health Organization (WHO), 1996, who.int/publications/i/item/WHOQOL-BREF, accessed June 25, 2025.*^6^ *WHO does not endorse any specific companies, products, or services.*
